# Impaired Oxidative Status Is Strongly Associated with Cardiovascular Risk Factors

**DOI:** 10.1155/2017/6480145

**Published:** 2017-12-12

**Authors:** E. Brunelli, D. La Russa, D. Pellegrino

**Affiliations:** Department of Biology, Ecology and Earth Sciences, University of Calabria, 87036 Cosenza, Italy

## Abstract

The main target of primary prevention is the identification of cardiovascular risk factors aimed at reducing of the adverse impact of modifiable factors, such as lifestyle and pharmacological treatments. In humans, an alteration of the oxidative status has been associated with several pathologies, including diabetes and cardiovascular diseases. However, the prognostic relevance of circulating oxidative stress biomarkers remains poorly understood. Our study explored, in a healthy population (*n* = 322), the relationship between oxidative status and cardiovascular risk factors. Here, we were successful in demonstrating that plasmatic oxidative status is significantly associated with traditional cardiovascular risk factors. We revealed a significant depletion in the efficacy of total plasma antioxidant barrier in high cardiovascular risk categories, and we confirmed an age-related alteration of oxidative status. The efficacy of total plasma antioxidant barrier is significantly depleted in relation to metabolic disorders. Interestingly, the cholesterol imbalance is the main factor in depleting the efficacy of total plasma antioxidant barrier. The oxidative status is also influenced by hypertension, and a slight increase in systolic blood pressure determines a highly significant effect. We showed that the first detectable event of a redox disturbance is the repairing intervention of the antioxidant barrier that is thus decreased as overutilized.

## 1. Introduction

Cardiovascular diseases (CVDs) are a group of diseases that share the principal risk factors and often the aetiology. The main manifestations of CVDs are coronary heart disease and stroke that represents the world's primary cause of death and disability and the most important cause of premature death, in agreement with the World Health Organization. CVDs represent a major health problem worldwide that causes a great public financial effort due to both inability to work and higher pharmaceutical expenditure. Therefore, for their broad and well-recognized importance, strategies to prevent CVDs should be considered as a priority for all citizens and healthcare systems.

The main target of primary prevention is the identification of cardiovascular risk factors aimed at reducing of the adverse impact of modifiable factors, such as lifestyle and pharmacological treatments. Furthermore, the evaluation of early and reliable risk factors can be used to identify high-risk subjects before the irreversible effects of the disease (early diagnosis). A growing number of scientific evidence suggests that effective prevention strategies are feasible and useful, also from the economic viewpoint [1].

A series of risk factors with pathogenic implication for CVDs have been identified and summarized in the Framingham study [2]. The main risk factors included smoking, hypertension, dyslipidemia, and diabetes. Over the years, several epidemiological studies validated the prediction models of cardiovascular diseases based on these risk factors, thus contributing to a steady decrease in CVD mortality [3], and the prediction models based on Framingham risk score are still used all over the world. Since the publication of results from Framingham study [2], other important predisposing factors with pathogenic implication for CVDs have been identified, including a high-fat diet, low physical activity, obesity, and genetic influences [4].

Currently, the ongoing studies are aimed at improving the risk algorithms through the individuation of new biomarkers strongly associated with CVDs (even if devoid of a direct relationship with these pathologies) also in order to define the appropriate preventive therapy of asymptomatic individuals [5, 6].

There are several clinical and experimental evidences supporting the hypothesis of a link between the oxidative status alteration and the development and progression of many health problems, such as neurodegenerative conditions, cardiovascular and inflammatory diseases, and cancer [7–9]. The predictive value of circulating oxidative stress biomarkers is poorly understood, despite the modified oxidative status has been associated with over 100 diseases. In particular, the ability of oxidative stress biomarkers to predict CVDs has been widely studied but remains largely unclear [10].

Oxidative stress is referred to the disproportion between free radicals and antioxidant system to counteract or detoxify their detrimental effects. The direct detection of free radicals is made complex by the nonspecificity and the high reactivity of these molecules. It takes, therefore, evaluating oxidative damage by measurement of secondary products, although the limited evidence that it reflects is oxidative status *in vivo* [11]. Epidemiological investigations have considered just a few of the numerous oxidant species as a biomarker relating them with cardiovascular dysfunctions, such as homocysteine, nitrosated tyrosines, and isoprostanes [12].

An alternative approach to investigate oxidative imbalance is the assessment of antioxidant enzymes (superoxide dismutase, catalase, and ascorbate peroxidase) and antioxidative defense, as well as nonenzymatic ascorbate, glutathione, flavonoids, tocopherols, and carotenoids [12, 13]. However, the predictive ability of these biomarkers and their usefulness to the definition of cardiovascular risk scores are underinvestigated. In the last years, several researchers are using two simple methods for detecting in vivo reactive oxygen species (ROS) using derivatives of reactive oxygen metabolites (dROMs) and biological antioxidant potential (BAP) [14–17]. For instance, in Japanese and Korean epidemiological trials, a significant correlation between oxidative balance and lifestyle-related diseases was found through these new methods [18, 19]. Hence, it is evident that there is a need for more extensive studies on large cohorts and under different clinical situations, including preclinical stages.

In view of this background, our research was designed to investigate, in a Mediterranean population, whether the oxidative balance is related to traditional cardiovascular risk factors. We evaluate, through a cross-sectional analysis on 322 healthy subjects, the global plasmatic oxidant/antioxidant ability by measuring reactive oxygen metabolite and biological antioxidant potential by photometric measurement. This study is of emerging interest in CVD research since the analysis of new biomarkers could improve the predictive role of CVD risk factors.

## 2. Subjects and Methods

### 2.1. Subjects

Our study involved 322 healthy Italian volunteers (work suitable) of both sexes and aged between 25 and 69 years (190 males, mean age: 51.42 ± 11.08 years and 132 female subjects, mean age: 46.11 ± 10.40 years) recruited from University of Calabria (UNICAL) staff during the annual visit performed by “UNICAL Prevention and Protection Service”. The volunteers were subjected to a “health check” by filling in a form (information on health status and lifestyle), by physical measurements (body mass index, systolic, and diastolic blood pressure), and by blood tests (blood glucose, lipoprotein panel, prooxidant, and antioxidant status). All subjects were studied in the morning and in a fasting state. Blood samples were taken from the antecubital vein and immediately centrifuged (2500*g* for 15 min at 4°C), and the plasma obtained was stored at 4°C until measurements (maximum 6 hours of venous blood collection). Baseline characteristics of the cohort are shown in Table 1.

### 2.2. Cardiovascular Risk Chart

The cardiovascular risk charts, based on the global absolute risk, are a simple and verified way of assessing the probability of experiencing a first major cardiovascular event (myocardial infarction or stroke) over the following ten years, by using the values of six risk factors: gender, diabetes, smoking, age, systolic blood pressure, and total serum cholesterol. When applied to the population from which they derive, they provide the best estimate of CVD risk. Therefore, in this study, we used Italian cardiovascular risk chart of The CUORE Project (http://www.cuore.iss.it). The risk charts are four: diabetic man, nondiabetic man, diabetic woman, and nondiabetic woman. For each of these four categories, the charts are further divided into smokers and nonsmokers, and the risk is calculated on the basis of age decade, serum cholesterol, and arterial pressure values. Six cardiovascular risk categories were constructed, called MCV (from I to VI): the CVD risk category indicates how many persons out of 100 with the same characteristics will fall ill over the next 10 years.

### 2.3. Oxidative Status and Biological Antioxidant Potential Measurements

Oxidative status and biological antioxidant potential determination were performed by using photometric measurement kits and a free radical analyzer system provided with spectrophotometric device reader (FREE Carpe Diem, Diacron International, Grosseto, Italy). All analyses were performed on ice-stored samples within maximum 6 hours of venous blood collection to prevent auto-oxidation phenomenon. We used Diacron reactive oxygen metabolite (dROM) and biological antioxidant potential (BAP) tests to evaluate plasma levels of reactive oxygen metabolites and antioxidant capacity. The dROM test helps to determine the oxidant ability of a plasma sample measuring the presence of reactive oxygen metabolites derivatives, in particular, hydroperoxides. By means of an appropriate acidic buffer, transition metal ions (essentially iron), originating by protein, are converted to alkoxy and peroxy radicals that react with hydroperoxides thus forming new radicals; aromatic amine (N,N-diethylparaphenylene-diamine) reacts with these new radicals originating a colored cation radical spectrophotometrically detectable at 505 nm [14, 15]. Results are expressed in Carratelli units (UC; 1 UC = 0.8 mg/L of hydrogen peroxide). The BAP test provides an overall measure of the biological antioxidant potential measuring the blood concentration of antioxidants (such as bilirubin, uric acid, vitamins C and E, and proteins) capable of reducing iron from ferric to the ferrous form; in fact, when the plasma is mixed with a colored solution (ferric chloride and thiocyanate), a decoloration occurs whose intensity is related to the ability of the plasma to reduce the ions of iron [16, 17]. The intensity of decoloration is spectrophotometrically detectable at 505 nm. Results are expressed in *μ*mol/L of the reduced ferric ions.

### 2.4. Statistical Analysis

Data have been analyzed using GraphPad/Prism version 5.01 statistical software (SAS Institute, Abacus Concept Inc., Berkeley, CA, USA). Differences between groups were examined using the unpaired *t*-test, or the Mann–Whitney test, or the Dunn's test, or the Kruskal-Wallis test, or the ANOVA test. A *p* value of < 0.05 was considered to be statistically significant. Data are expressed as the mean ± standard deviation.

### 2.5. Ethics Statement

All investigations have been conducted according to the Declaration of Helsinki principles and have been approved by Local Ethical Committee (n°8/2016, Regione Calabria, Sezione Area Nord). All subjects have provided written informed consent that, as guarantor, is retained by the corresponding author.

## 3. Results

Our study population consists of 322 subjects (190 males and 132 females) aged between 25 and 69 years (25–39, *n* = 79; 40–49, *n* = 71; 50–59, *n* = 107; and 60–69, *n* = 65).

Baseline characteristics of the cohort are shown in Table 1. These data are comparable to the results of the second population survey of Cardiovascular Epidemiologic Observatory (The CUORE Project—Istituto Superiore Sanità—Italy) relative to a population sample from Calabria monitored in the period 2008–2012 (http://www.cuore.iss.it/eng/factors/south.asp). In the whole sample, oxidative status and antioxidant barrier efficacy values are the following: dROM test = 333.80 ± 72.94 UC and BAP test = 1968.96 ± 412.21 *μ*mol/L. By suitable statistical tools, we analyzed the trend of both oxidative status and antioxidant barrier efficacy in order to identify possible correlations with MCV and traditional cardiovascular risk factors: gender, diabetes, smoking, age, systolic blood pressure, and total serum cholesterol. We also considered further determinants that may predispose to cardiovascular risk as menopausal status, obesity, and the ratio of total cholesterol to HDL (high-density lipoproteins).

### 3.1. MCV

**Figure 1:**
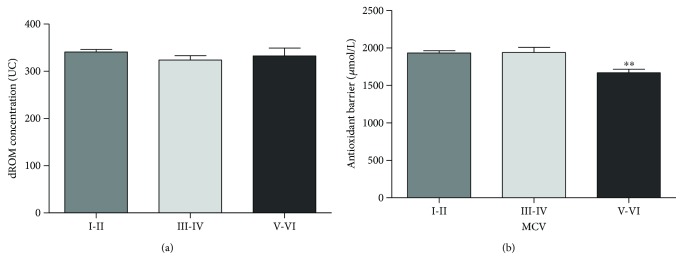
Values of dROM (a) and BAP (b) tests by MCV (data are expressed as mean ± SE; Dunn's test, ^∗∗^*p* < 0.005).

We calculated the total CVD risk of our cohort using the Italian cardiovascular risk chart of The CUORE Project. MCV category (from I to VI) has been assigned to subjects aged between 40 and 69 years (*n* = 243) based on parameters described in http://www.cuore.iss.it/eng/assessment/risk_assessment.asp. We analyzed the trend of both oxidative status and antioxidant barrier efficacy by comparing subjects with low (MCV I-II; *n* = 180) medium (MCV III-IV; *n* = 45), and high (MCV V-VI; *n* = 18) total CVD risk. We found no significant differences in ROM values between MCV categories (Figure 1(a)), while we showed a significant decrease in antioxidant barrier efficacy in high (MCV V-VI) CVD risk categories (Dunn's test, *p* < 0, 005; Figure 1(b)).

### 3.2. Cardiovascular Risk Factor: Gender

**Figure 2:**
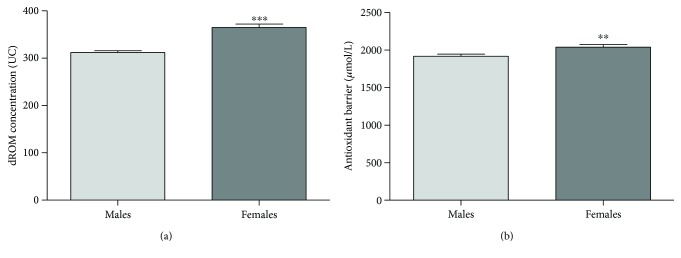
Values of dROM (a) and BAP (b) tests by gender (data are expressed as mean ± SE; Mann–Whitney test, ^∗∗∗^*p* < 0.0001; ^∗∗^*p* < 0.005).

We found a significant difference between males (*n* = 190) and females (*n* = 132) in the values of both oxidative status (Mann–Whitney test, *p* < 0.0001; Figure 2(a)) and antioxidant barrier efficacy (Mann–Whitney test, *p* < 0.001; Figure 2(b)). In particular, females present high ROM (364.70 ± 85.96 UC) and BAP (2035.74 ± 412.28 *μ*mol/L) values, while males show ROM values close to the normal values (312.00 ± 52.30 UC) and low BAP values (1915.03 ± 406.64 *μ*mol/L). Within the female group, no significant difference was observed in premenopausal (*n* = 87) and postmenopausal (*n* = 45) subjects (ROM values: premenopausal 366.49 ± 50.83 UC and postmenopausal 361.22 ± 45.92 UC; BAP values: premenopausal 2079.32 ± 441.73 *μ*mol/L and postmenopausal 1951.49 ± 337.15 *μ*mol/L).

### 3.3. Cardiovascular Risk Factor: Diabetes and Obesity

**Figure 3:**
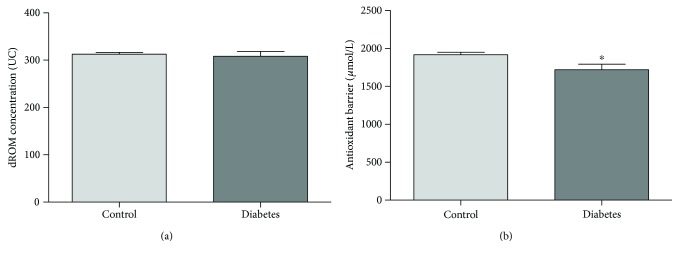
Values of dROM (a) and BAP (b) tests by diabetes status (data are expressed as mean ± SE; Mann–Whitney test, ^∗^*p* < 0.05).

**Figure 4:**
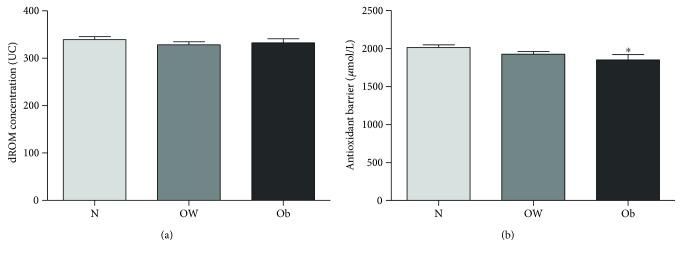
Values of dROM (a) and BAP (b) tests by BMI (data are expressed as mean ± SE; Kruskal-Wallis test, ^∗^*p* < 0.025).

In our study population, only 14 people, all males, had a diagnosis of diabetes and were undergoing insulin or oral hypoglycemic agent treatment. Therefore, we analyzed the values of oxidative status and antioxidant barrier efficacy by comparing nondiabetic males (*n* = 176) with respect to diabetic males (*n* = 14). Despite the small sample size, we found a significant decrease in antioxidant barrier efficacy in diabetic subjects (Mann–Whitney test, *p* < 0.05; Figure 3(b)) while no differences were evidenced in oxidative status (Figure 3(a)). Body mass index (BMI) is a simple index of weight-for-height that is commonly used to classify underweight, overweight, and obesity in adults. To analyze the trend of both oxidative status and antioxidant barrier efficacy in relation to BMI, we divided our study population according to this international classification: underweight (UW, <18.50 BMI, *n* = 0); normal range (N, 18.50–24.99 BMI, *n* = 150); overweight (OW, ≥25.00 BMI, *n* = 126); and obese (Ob, ≥30.00 BMI, *n* = 35). Our results showed that there were no differences in ROM values among N, OW, and Ob groups (Figure 4(a)) as a decrease in antioxidant barrier efficacy can be observed already in OW subjects, reduction statistically significant in Ob subjects (Figure 4(b); Kruskal-Wallis test, ^∗^*p* < 0.025).

### 3.4. Cardiovascular Risk Factor: Smoking

Concerning smoking habits, equally distributed between sex (17%), we found no significant differences between nonsmoker (*n* = 267) and smoker (*n* = 55) subjects in the values of both oxidative status and efficacy of antioxidant barrier (ROM values: nonsmokers 335.03 ± 76.23 UC and smokers 329.05 ± 55.06 UC; BAP values: nonsmokers 1968.14 ± 415.10 *μ*mol/L and smokers 1944.09 ± 405.46 *μ*mol/L). However, it should be noted that the smoker sample consists mainly of moderate smokers (less than ten cigarettes daily).

### 3.5. Cardiovascular Risk Factor: Age

**Figure 5:**
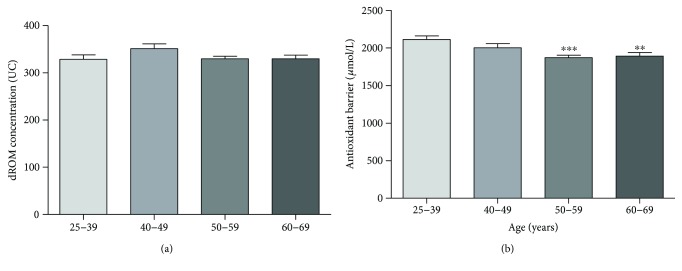
Values of dROM (a) and BAP (b) tests by age (data are expressed as mean ± SE; Kruskal-Wallis test, ^∗∗∗^*p* < 0.0005; ^∗∗^*p* < 0.005).

We divided our study population into four age groups: the first group (25–39 years, *n* = 78) not provided for cardiovascular risk chart and used herein as controls and three groups according to the age decades of cardiovascular risk chart (40–49 years, *n* = 71; 50–59 years, *n* = 105; and 60–69 years, *n* = 63). The ROM values remain at a constant level in all groups (Figure 5(a)), while a constant reduction of antioxidant barrier efficacy was observed with increasing age (Figure 5(b)). This reduction is remarkable and highly statistically significant starting from 50 years of age (Kruskal-Wallis test, 50–59 years *p* < 0.0005 and 60–69 years *p* < 0.005).

### 3.6. Cardiovascular Risk Factor: Systolic Blood Pressure

**Figure 6:**
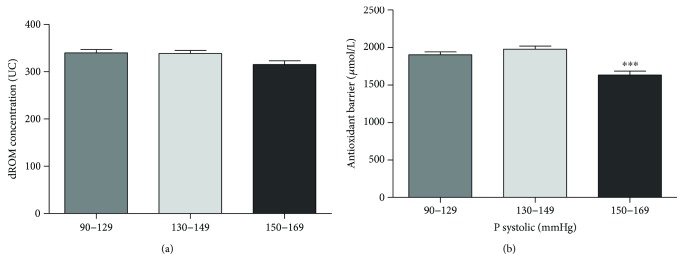
Values of dROM (a) and BAP (b) tests by systolic pressure (data are expressed as mean ± SD; Kruskal-Wallis test, ^∗∗∗^*p* < 0.0005).

To analyze the systolic blood pressure values in relation to oxidative status and antioxidant barrier efficacy, we divided our study population into three groups according to the range of cardiovascular risk chart (90–129 mmHg, *n* = 174; 130–149 mmHg, *n* = 103; and 150–169 mmHg, *n* = 26). The ROM values remain at a constant level in all groups (Figure 6(a)), while a statistically significant reduction of antioxidant barrier efficacy was observed starting from 150 mmHg of systolic blood pressure values (Figure 6(b); Kruskal-Wallis test, *p* < 0.0005).

### 3.7. Cardiovascular Risk Factor: Total Serum Cholesterol and Ratio of Total to HDL

**Figure 7:**
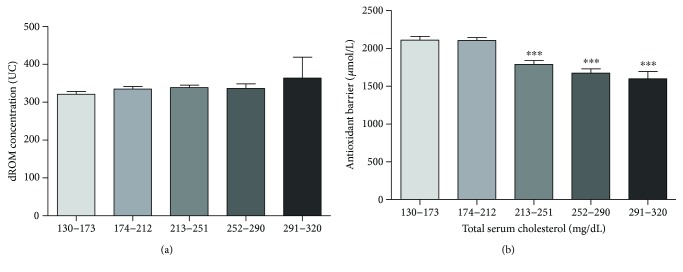
Values of dROM (a) and BAP (b) tests by total serum cholesterol (data are expressed as mean ± SD; Kruskal-Wallis test, ^∗∗∗^*p* < 0.0001).

**Figure 8:**
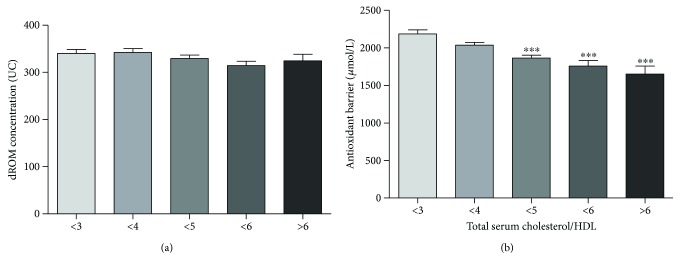
Values of dROM (a) and BAP (b) tests by total serum cholesterol/HDL (data are expressed as mean ± SD; Kruskal-Wallis test, ^∗∗∗^*p* < 0.0001).

We analyzed both total serum cholesterol values and the ratio of total to HDL fraction in relation to oxidative status and antioxidant barrier efficacy. In relation to total serum cholesterol values, we divided our cohort in five groups according to the range of cardiovascular risk chart (130–173 mg/dL, *n* = 56; 174–212 mg/dL, *n* = 124; 213–251 mg/dL, *n* = 91; 252–290 mg/dL, *n* = 30; and 291–320 mg/dL, *n* = 9). The ROM values remain at a constant level in all groups (Figure 7(a)), while a constant reduction of antioxidant barrier efficacy was observed with increasing total serum cholesterol values (Figure 7(b)). This reduction is remarkable and highly statistically significant starting from 213 mg/dL of total serum cholesterol (Kruskal-Wallis test, *p* < 0.0001). Concerning the ratio of total cholesterol to HDL fraction, we divided the study population into five groups: ratio of less than 3 (<3, *n* = 59); ratio of less than 4 (<4, *n* = 107); ratio of less than 5 (<5, *n* = 93); ratio of less than 6 (<6, *n* = 33); and ratio greater than 6 (>6, *n* = 7). Even in this case, the ROM values remain at a constant level in all groups (Figure 8(a)), while a remarkable and highly statistically significant reduction of antioxidant barrier efficacy was observed with increasing ratio of total cholesterol to HDL fraction (Figure 8(b); Kruskal-Wallis test, *p* < 0.0001).

## 4. Discussion

Our research focused on identifying the putative relationship between oxidative imbalance and cardiovascular risk factors through a cross-sectional analysis on a large healthy population. We clearly showed that the oxidative status is significantly associated with MCV, diabetes, obesity, age, high systolic blood pressure, serum cholesterol, and total cholesterol/HDL. In particular, we reported, for the first time, that the early warning alteration at a systemic level is the reduction of antioxidant capacity.

It is noteworthy that these results have been achieved using a noninvasive method to detect total plasma redox balance, which is of particular importance when analyzing the healthy subject. The analysis of the overall redox balance does not identify the impaired system/s, but it provides an evaluation of the imbalance induced by the alteration of individual parameters.

To the best of our knowledge, this is the first cross-sectional study that investigated, on a healthy cohort, the oxidative status taking into account all the cardiovascular risk factors. Although a direct causality cannot be inferred from such kind of correlative investigations, our data provide an important contribution to understanding the cross-talk between oxidative imbalance and cardiovascular risk factors also representing a point of departure to address further investigation.

### 4.1. MCV

Global comparative risk assessment and associated health effect studies have estimated that hundreds of thousands or millions of CVD deaths are attributable to established CVD risk factors and other putative, emerging, risk factors that are the subject of extensive research. In particular, several studies suggested a positive correlation between CVDs/CVD risk factors and an increased oxidative stress [7–9]. However, information on healthy populations are scarce, and most of the available data derive from trials conducted on subjects with very high cardiovascular risk [20]. In these situations, it becomes difficult to establish a clear understanding of the relative influence of the different factors in determining oxidative imbalance and then to evaluate the synergic or independent action of CVD risk factors.

Because traditional risk factors account for only a fraction of CVDs, the importance of alternate and additional predictors is evident [21, 22]. Our results, exploring the oxidative status of healthy subjects (without previous cardiovascular events), show a significant association between the high cardiovascular risk (MCV V-VI) and the depletion in the efficacy of total plasma antioxidant barrier despite the normal values of oxidative status. Recently, some authors found a significant correlation between ROM values and age [18] or lipid profile [19], but our study remains the only considering overall CVD risk factors.

Antioxidant deficiencies may be the result of a decreased antioxidant intake, a reduced synthesis of endogenous enzymes, or an increased antioxidant utilization [22]. Since the antioxidant species are numerous and they operate synergistically, evaluating the activity of each antioxidant species may underestimate the association among different effects and probably do not reflect the physiological conditions. Moreover, for each antioxidant compound, a specific test is needed thus making the evaluation of antioxidant capacity extremely complex [23, 24].

We recognize that there are currently no validated methods for quantifying the oxidative status but certainly, the total antioxidant capacity is indicative of both organism antioxidant protection and oxidative stress amount.

### 4.2. Gender

According to our previous findings [25], we showed that the oxidative status was significantly higher in females than in males and we also demonstrate that this result is not related to the level of circulating hormones since no differences have been detected between pre- and postmenopausal women. The physiological significance of this gender-related difference remains unclear, and research on both animals and humans have shown contrasting results ([25] and references therein). Another interesting finding in our study was the more effective antioxidant barrier detected in females compared to males suggesting that, in healthy subjects, the altered oxidative status is balanced by an enhancement in the antioxidant barrier effectiveness.

### 4.3. Diabetes and Obesity

Obesity is an important cause of CVDs, and it promotes a cluster of risk factors including dyslipidemia, type 2 diabetes, and hypertension [26]. Several pieces of evidence support the role of oxidative stress in obesity and diabetes metabolic perturbations (and subsequent cardiovascular pathogenesis) [27, 28]. The proinflammatory and prooxidant effects of an increased adiposity represent a potential link between obesity and CVDs, even in the absence of other risk factors [29, 30]. The positive association between indices of obesity and oxidative stress biomarkers is well acknowledged [20], but the underlying mechanisms are complex and not yet fully identified. In the obese-diabetic patients, the excessive uric acid has been shown to induce CVDs through the generation of ROS and subsequent endothelial dysfunction [31]. Recent studies have emphasized the importance of antioxidant defense in type 2 diabetes patients. In these subjects, the excessive ROS stimulation leads to a progressive deterioration of the antioxidant system that tends to crumble [31]. In our cohort, we found a significant decrease in antioxidant barrier efficacy in both diabetic and obese subjects; these results contribute to emphasize the importance of antioxidant barrier effectiveness in countering the deleterious effects of ROS overproduction.

### 4.4. Smoking

Smoking is an important risk factor for cardiovascular disease development. Cigarette smoke is a complex mixture of chemical compounds, containing many free radicals and oxidants [32, 33], and it can be associated with oxidative stress in smokers [34, 35]. It has also been highlighted a direct correlation between oxidative index and number of cigarettes smoked [36]. In our cohort, we found no significant differences in the oxidative status between nonsmokers and smokers. However, the smoker sample in our study was small and consisted mainly of moderate smokers (less than ten cigarettes daily). Moreover, the ex-smokers were very few and all of them had stopped smoking for more than ten years.

### 4.5. Age

The oxidative stress theory of aging postulates that reactive oxygen species play a key role in the aging process through an age-related accumulation of oxidative damages in macromolecules, resulting in a progressive loss of cellular function and senescence [37]. Over the past two decades, several lines of evidence supported this theory [38] and a number of experimental studies, in both humans and animals, showed a linear correlation between age and oxidative stress [39]. In our study, we detected no difference in ROM values in the different age groups whereas a regular reduction of antioxidant barrier efficacy was observed with increasing age. It is well known that antioxidants delay or protect against the damage produced by free radical reactions and are consumed during this process. In fact, global antioxidant status is even used to indirectly evaluate free radical activity [24].

### 4.6. Blood Pressure

Endothelial dysfunction, the initial stage in the pathogenesis of several cardiovascular diseases including hypertension, is associated with increased vascular ROS production, oxidative stress, and vascular inflammation [40]. Clinical studies, in patients with essential hypertension, demonstrated that systolic and diastolic blood pressure correlate positively with oxidative stress biomarkers [41, 42], and similar results have been found in rats [43]. Direct measurements of ROS vascular production in hypertensive subjects demonstrated higher levels of O_2_ and H_2_O_2_ and an enhanced angiotensin II-stimulated redox signaling compared with cells from normotensive counterparts [44, 45]. In our study, we divided the healthy population into three groups, according to the range of cardiovascular risk charts (90–129 mmHg; 130–149 mmHg; and 150–169 mmHg), and we did not observe any change in the ROM values. On the contrary, we found a statistically significant reduction of antioxidant barrier efficacy in the group with systolic blood pressure higher than 150 mmHg. Several observational studies have reported an inverse relationship between blood pressure and antioxidant levels [46–48]. A decreased antioxidant activity and reduced levels of ROS scavengers might contribute to induce oxidative stress in hypertensive subjects, but also an increase in vascular ROS production has been hypothesized to reduce the antioxidant efficacy [8].

### 4.7. Lipidic Profile

Lipid metabolism disorders are associated with the overproduction of reactive oxygen species and have been shown to affect the antioxidant status and the lipoprotein levels in different organs [49, 50]. Dyslipidemia in combination with endothelial damage is a crucial event in the most common pathological processes underlying CVDs [51, 52]. In addition, endothelial dysfunction can be started/supported by several factors, including an excess of ROS and the exposure to harmful agents such as oxidized LDL [53]. In our study, we did not find a significant relationship between changes in oxidative status and total cholesterol values whereas a regular and significant reduction of antioxidant barrier efficacy was observed in subjects with pathological cholesterol values (total serum cholesterol values > 213 mg/dL).

We observed a similar result by relating oxidative status with total cholesterol/HDL cholesterol ratio. According to evidence from large observational studies, total cholesterol/HDL cholesterol ratio seems to be a more powerful risk predictor than isolated parameters used independently ([54] and references therein). Indeed, both diagnosis and treatment of dyslipidemia, including instruments for calculating cardiovascular risk factors, nowadays include the lipoprotein ratios that, in view of the evidence-based results, present greater predictive power [54].

Interestingly, in a recent paper, Yagi and colleagues [55] demonstrated that BAP was strongly correlated with carotid artery IMT suggesting that it may be considered a suitable risk marker for carotid atherosclerosis; moreover, they postulate that the measurements of BAP may be superior to the measurements of glutathione peroxidase, superoxide dismutase, catalase, and total antioxidant status for the assessment of antioxidant potential. Our results emphasize that the first detectable event of a redox disturbance is the repairing intervention of the antioxidant barrier that is thus decreased as overutilized.

## 5. Conclusion

In the present study, we showed through a cross-sectional analysis on a large healthy population that a reduced antioxidant capacity is significantly associated with cardiovascular risk factors. In epidemiological studies, the magnitude of the cohort is a key factor for the validity of the results. Our numbers reach an average value; therefore, our research can be considered as a pilot study and as the first application of a protocol aimed to verify the validity of the experimental design. Moreover, we assessed the oxidative status through indirect determinations thus providing an overall measure of many oxidants/antioxidants, also without identifying the molecules involved in the perturbation of normal homeostasis. The assessment of both validity and reproducibility of such indirect determinations is important as they represent an analytical tool not many invasive and easy to perform which allows an application on a large scale. Further studies are needed to clarify better how these new putative biomarkers and the traditional risk factors are related and how they can improve the prediction of cardiovascular risk.

## Figures and Tables

**Table 1:** Baseline characteristics of the cohort (*n* = 322; data are expressed as mean ± SD).

		Normal range
Age (years)	49.24 ± 11.10	
BMI (body weight/height^2^)	25.95 ± 9.19	18.50–24.99
Systolic blood pressure (mmHg)	123.07 ± 16.08	<120
Diastolic blood pressure (mmHg)	76.31 ± 9.44	<80
Blood glucose (mg/dL)	97.22 ± 22.54	70–99
Total cholesterol (mg/dL)	206.12 ± 40.45	<240
HDL cholesterol (mg/dL)	54.76 ± 14.37	>60
LDL cholesterol (mg/dL)	132.03 ± 32.22	<115
Triglycerides (mg/dL)	119.01 ± 61.76	<150
Smokers	55 (17%)	
